# Acute Stroke and Limb Ischemia Secondary to Catastrophic Massive Intracardiac Thrombus in a 40-Year-Old Patient With Dilated Cardiomyopathy

**DOI:** 10.4021/cr142w

**Published:** 2012-01-20

**Authors:** Gi Jung Jeon, Bong Gun Song, Yong Hwan Park, Gu Hyun Kang, Woo Jung Chun, Ju Hyeon Oh

**Affiliations:** a Division of Cardiology, Cardiac and Vascular Center, Department of Medicine, Samsung Changwon Hospital, Sungkyunkwan University School of Medicine, Changwon, Korea

**Keywords:** Dilated cardiomypathy, Thrombus, Thromboembolism, Echocardiography

## Abstract

Dilated cardiomyopathy has been associated with left ventricular (LV) thrombosis which leads to substantial morbidity and mortality as a site for systemic emboli. We report an interesting case of a stroke and acute limb ischemia secondary to a large mobile pedunculated LV thrombus in 40-year-old patient with dilated cardiomyopathy.

## Introduction

Left ventricular (LV) thrombi are seen in the setting of myocardial infarction, LV aneurysm, and dilated cardiomyopathy [1]. Identification of LV thrombi in dilated cardiomyopathy is of major importance since it can predispose to catastrophic arterial embolic events [1, 2]. We report an interesting case of a stroke and acute limb ischaemia secondary to a large mobile pedunculated LV thrombus in 40-year-old patient with dilated cardiomyopathy.

## Case Report

A 40-year-old male was referred to our hospital due to dyspnea, right facial and limb weakness, and both lower limb pain. He had no history of smoking, hypertension, diabetes mellitus, hyperlipidemia, alcohol intake or recent viral infection and had no medicine. There was no family history of cardiac disease. He underwent orthopedic surgery for both leg fractures due to traffic accident one year age. On arrival, He was tachypnoeic, tachycardic and had a Glasgow Coma Scare of 15 with oxygenation saturations of 85% on air. On examination his both lower limbs were cold and pulseless. Neurologic examination revealed mild hemiparesis on the right side. Initial chest radiography showed no abnormalities except for mild cardiomegaly (cardiothoracic ratio was 0.65). Electrocardiography (ECG) showed normal sinus rhythm with diffuse non-specific ST segmental changes. His laboratory findings showed elevated levels of brain natriuretic peptide (2135 pg/mL), blood urea nitrogen (25.0 mg/dL), Serum creatinine (1.8 mg/dL), CK-MB (3.1 ng/mL), and tropinin-I (0.59 ng/mL). All hematological data as well as markers of thrombosis and fibrinolysis were within normal limits: hemoglobin was 14.6 g/dL, platelet count was 317 × 10^3^ /µL, prothrombin time-international normalized ratio was 0.89, activated partial thromboplatin time was 28.1 seconds, anti-thrombin-III was 64%, D-dimer was 0.5µg/mL, fibrin degradation product was 1.0 µg/mL, protein C activity was 75%, protein S activity was 80%, IgG and IgM anticardiolipin antibodies were negative, and lupus anticoagulant was negative.

**Figure 1:**
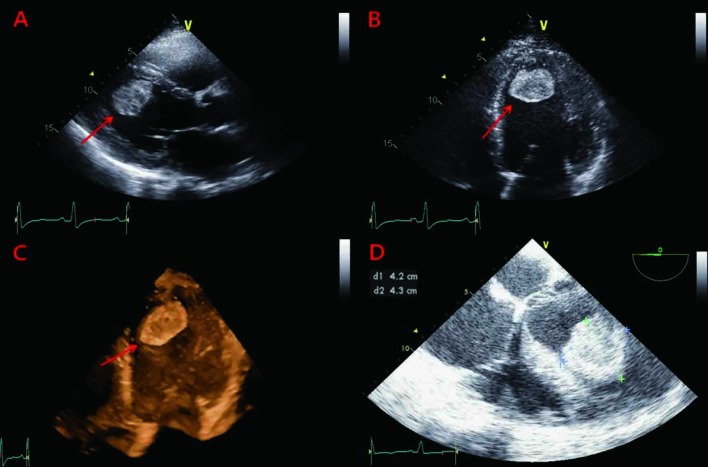
Transthoracic (A-C) and transesophageal (D) echocardiograms showed severe global LV systolic dysfunction and dilatation with a large mobile pedunculated mass in the LV apex.

**Figure 2:**
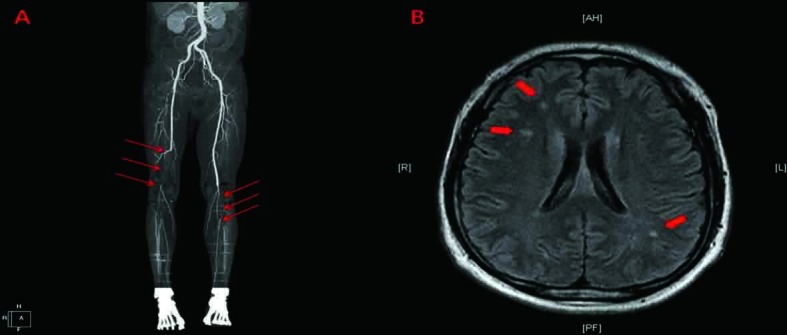
Computed tomography angiography and brain magnetic resonance imaging showed obstructions of right femoral and popliteal artery and left popliteal artery (A) and spotty ischemic brain lesions (B).

**Figure 3:**
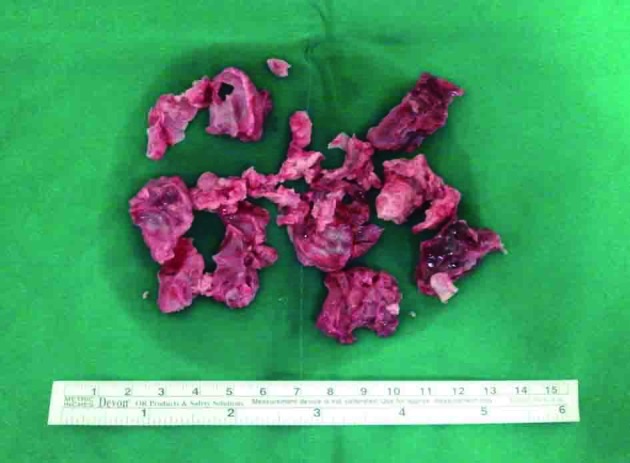
The gross examinations of specimen removed from LV apex showed thrombus with multiple fragments of deep reddish blood clot-like material.

Transthoracic and transesophageal echocardiograms (done at operation room) revealed severe global LV systolic dysfunction (left ventricular ejection fraction [LVEF] of 19%) and dilatation (LV diameter of 65 cm in systole) with ovoid shaped hyperechoic mass-like lesion in LV apex (Fig. 1). A large mobile pedunculated mass measuring 4.3 × 4.2 cm was attached with a narrow stalk to the apical segment and the protruding into the LV cavity (Fig. 1). The patient was thought to have idiopathic cardiomyopathy in the absence of other detectable secondary causes as well as coronary risk factors. In the presence of severe LV systolic dysfunction, the mass-like lesion was suspected of being a thrombus. However, the possibility of a cardiac tumor could not be excluded. Brain magnetic resonance imaging and computed tomography angiography were performed for evaluation of right hemiparesis, coldness and pulseless of both lower limbs and showed spotty ischemic brain lesions and obstructions of right femoral and popliteal artery and left popliteal artery, respectively (Fig. 2 A, B). In view of the higher risk of systemic embolization and the uncertainty of its nature, surgical removal of the mass was recommended. Low-molecular-weight heparin was started and he subsequently underwent urgent surgical removal for the mass via mitral valve approach concomitant with embolectomy for thromboemboli of lower limbs. The gross and microscopic examinations of specimen removed from LV apex revealed thrombus with multiple fragments of deep reddish blood clot-like material (Fig. 3). After his clinical and laboratory findings were stabilized coronary angiography was performed, which revealed an insignificant stenosis (5%) on right coronary artery. At postoperative seventh day, he was discharged with beta blocker, ACE inhibitor and oral warfarin and then started outpatient rehabilitation. At 1-year follow-up he is doing well. His cardiac function was normalized (LVEF of 59%; LV diameter of 48 cm in systole) without recurrence of thrombus.

## Discussion

Intracardiac thrombus has been associated with many diseases and clinical states, although cardiac impairment is commonly present and associated with stroke and thromboembolic risk [1, 2]. The common causes of thrombus formation in the left ventricle remain ischemic cardiomyopathy, LV aneurysm, and dilated cardiomyopathy [1].

The incidence of LV thrombus in patients with cardiomyopathy has been reported in the literature as 11 - 44% [3]. The morphologic characteristics of the thrombus together with the LV contractility are the primary determinants of embolic risk [3, 4]. A pedunculated thrombus that is connected to the ventricular wall or septum by a narrow stalk and that moves throughout the cardiac cycle is an unusual type of LV thrombus compared with mural thrombus [4]. Such thrombi have an especially high tendency to embolize despite adequate anticoagulation [4].

The definitive treatment of LV thrombi is still controversial. Over the last 30 years, the primary therapeutic options for such thrombi have included thrombectomy, anticoagulation, or thrombolysis [1, 2]. The benefits of anticoagulants in patients with dilated cardiomyopathy have been reported in many studies, with a reduction in thromboembolic events or resolution of thrombus on echocardiography [5-7]. Kyrle et al [6] reported that arterial or pulmonary thromboembolism occurred in 17 of 38 patients with dilated cardiomyopathy before starting oral anticoagulants, but no thromboembolic episodes occurred whilst on anticoagulants. In the study by Fuster et al [7], none of the patients with dilated cardiomyopathy who were taking anticoagulants had a thromboembolic episode, compared to 14 thromboembolic events in the 104 patients who were not anticoagulated. High dose intravenous heparin or low molecular-weight heparin may effectively treat the mobile thrombi that protrude into the LV cavity [8, 9]. Rester et al reported successful lysis of a pedunculated, mobile LV thrombus with recombinant tissue plasminogen activator in a patient with peripartum cardiomyopathy and evidence of systemic embolization [10]. However, the risk of hemorrhagic or emboli complications may be unacceptably high [10].

Surgical removal is generally recommended for mobile and pedunculated thrombi because they have a significantly higher risk of systemic embolization and it has usually been carried out by ventriculotomy [4]. However, ventricular wall incision may cause deterioration of LV function and potentially induce ventricular arrhythmia [4, 11]. Transaortic video assisted removal of a LV thrombus, and also the trans-left atrial appendage and mitral valve approach, can provide good LV visualization [11, 12]. However, there are serious potential complications related to video assisted cardioscopy [12]. Transatrial method of thrombectomy also can allow avoidance of a ventriculotomy [11].

Our patient had a mobile and pedunculated LV mass which was thought to be a thrombus secondary to an idiopathic cardiomyopathy. Because of its narrow stalk attachment to the wall and concomittant systemic embolization, the potential risk for another systemic embolization was thought to be extremely high and thus, surgical removal was recommended via mitral valve approach to avoid deterioration of LV function.
